# Revisiting Cancer Stem Cells as the Origin of Cancer-Associated Cells in the Tumor Microenvironment: A Hypothetical View from the Potential of iPSCs

**DOI:** 10.3390/cancers12040879

**Published:** 2020-04-04

**Authors:** Amira Osman, Said M. Afify, Ghmkin Hassan, Xiaoying Fu, Akimasa Seno, Masaharu Seno

**Affiliations:** 1 Laboratory of Nano-Biotechnology, Graduate School of Interdisciplinary Science and Engineering in Health Systems, Okayama University, Okayama 700-8530, Japan; mero.osman@med.kfs.edu.eg (A.O.); pthz2c4o@s.okayama-u.ac.jp (G.H.); pru57big@okayama-u.ac.jp (X.F.); aseno@okayama-u.ac.jp (A.S.); 2 Department of Histology, Faculty of Medicine, Kafrelsheikh University, Kafr Elsheikh 33511, Egypt; 3 Department of Medical Bioengineering, Graduate School of Natural Science and Technology, Okayama University, Okayama 700-8530, Japan; saidafify@s.okayama-u.ac.jp; 4 Division of Biochemistry, Chemistry Department, Faculty of Science, Menoufia University, Shebin El Koum-Menoufia 32511, Egypt; 5 Department of Microbiology and Biochemistry, Faculty of Pharmacy, Damascus University, Damascus 10769, Syria; 6 Department of Pathology, Tianjin University of Traditional Chinese Medicine, Tianjin 300193, China; 7 Okayama University Research Laboratory of Stem Cell Engineering in Detroit, IBio, Wayne State University, Detroit, MI 48202, USA

**Keywords:** CAFs, TECs, TAAs, TAMs, CSCs

## Abstract

The tumor microenvironment (TME) has an essential role in tumor initiation and development. Tumor cells are considered to actively create their microenvironment during tumorigenesis and tumor development. The TME contains multiple types of stromal cells, cancer-associated fibroblasts (CAFs), Tumor endothelial cells (TECs), tumor-associated adipocytes (TAAs), tumor-associated macrophages (TAMs) and others. These cells work together and with the extracellular matrix (ECM) and many other factors to coordinately contribute to tumor growth and maintenance. Although the types and functions of TME cells are well understood, the origin of these cells is still obscure. Many scientists have tried to demonstrate the origin of these cells. Some researchers postulated that TME cells originated from surrounding normal tissues, and others demonstrated that the origin is cancer cells. Recent evidence demonstrates that cancer stem cells (CSCs) have differentiation abilities to generate the original lineage cells for promoting tumor growth and metastasis. The differentiation of CSCs into tumor stromal cells provides a new dimension that explains tumor heterogeneity. Using induced pluripotent stem cells (iPSCs), our group postulates that CSCs could be one of the key sources of CAFs, TECs, TAAs, and TAMs as well as the descendants, which support the self-renewal potential of the cells and exhibit heterogeneity. In this review, we summarize TME components, their interactions within the TME and their insight into cancer therapy. Especially, we focus on the TME cells and their possible origin and also discuss the multi-lineage differentiation potentials of CSCs exploiting iPSCs to create a society of cells in cancer tissues including TME.

## 1. Introduction

Cancer stem cells (CSCs), identified as cancer-originating cells, are responsible for the maintenance and development of malignant tumors being defined by the potentials of self-renewal, differentiation, and tumorigenicity. The development of CSCs is generally considered to be regulated by genetic and epigenetic changes resulting in tumorigenic abilities, cytoplasmic signal transduction and metastasis [1]. Accumulating evidence has revealed that CSCs have a pluripotent differentiation ability like normal stem cells [2]. Moreover, CSCs have the potential to transdifferentiate into vascular endothelial cells and pericytes in vitro and in vivo [3,4]. Furthermore, several differentiated cells have been directly reprogrammed from one cell type into another with the induction of potent transcription factors [5].

Another study also supports that Osteopontin induces mesenchymal stem cells in the tumor microenvironment (TME) to differentiate into cancer-associated fibroblasts (CAFs), which promotes cancer development and can be stimulated to release periostin in the metastatic microenvironment [6,7].

TME has a vital role in cancer initiation and progression. During recent decades, the focus of cancer research has widened from the malignant tumor cells themselves to the TME and the complicated interactions between the host stroma and tumor cells [8,9]. The TME displays many similarities with the normal wound microenvironment, including angiogenesis, infiltration of fibroblasts and immune cells and widespread remodeling of extracellular matrix (ECM) [10]. Tumor bulk is heterogeneous in their composition. Recent studies have indicated that TME arises from at least six distinct cellular origins: fibroblasts [11], endothelial cells, pericytes, bone-marrow-derived mesenchymal stem cells (MSCs) [12], adipocytes [13], macrophages [14], and other immune cells [15]. Many substantial pieces of evidence indicate cellular transdifferentiation within the TME, both from tumor cells to stromal cells and from stromal cells to stromal cells such as fibroblast transdifferentiation into activated myofibroblast during the formation of tumor stroma [16]. Evidence has been suggested that this phenomenon may be the transdifferentiation [17] or differentiation events, according to the microenvironments. In certain conditions, pericyte can be transdifferentiated into tumor-associated stromal cells [18]. Another example, evidence indicates that cancer cells can be transdifferentiated into stromal cells to promote tumor growth [19].

Chronic inflammation has been hypothesized to stimulate the generation of CSCs. The cancer-inducing niche should, therefore, be developed from chronic conditions stimulating normal stem cells or progenitor cells to convert into CSCs, which are potent to differentiate into the phenotype of cancer cells. Once CSCs develop, the CSC niche with/without the cancer-inducing niche will provide a suitable microenvironment for sustaining CSCs, which in turn develop malignant tumors. The phenotype of the malignant tumor seems to depend not only on the tissue-specific microenvironment but on the niche of the CSCs, as previously reported [20]. We have originally demonstrated that iPSCs can acquire CSC characters when exposed to the conditioned medium derived from different cancer cell lines expressing various growth factors, cytokines, chemokines and so on [21]. CSCs will then establish their niche by themselves together with their progenies [22]. The cells in the CSC niche not only maintain CSCs and tumor-associated cells but also produce factors promoting invasion, metastasis, and angiogenesis. The components of CSC niche are typically considered as CAFs, tumor-associated macrophages (TAMs), tumor-associated neutrophils, MSCs as well as CSCs [23]. Among these cells, cell-to–cell communications should be made via cell-mediated adhesion, soluble factors and exosomes with their critical roles.

**Figure 1:**
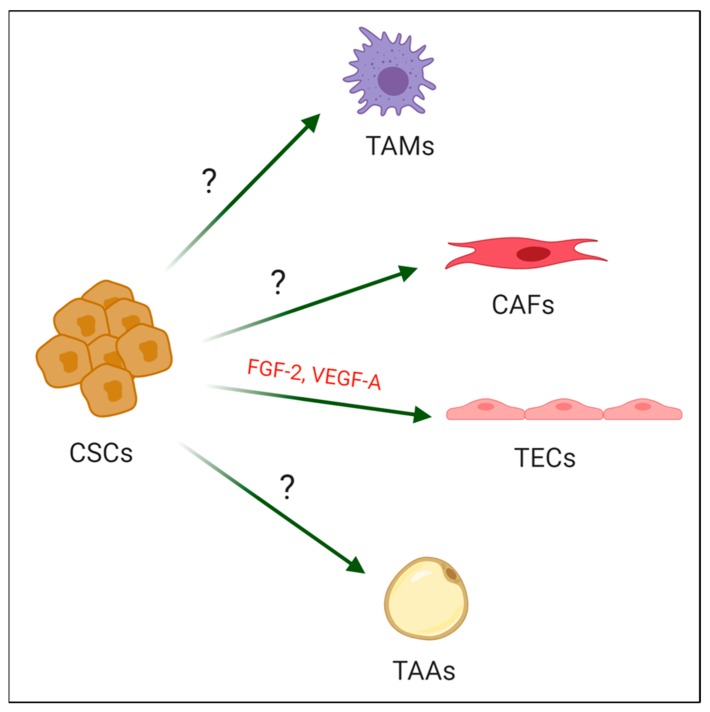
A schematic illustration showing cancer stem cells’ (CSCs) differentiation into tumor stromal cells, Tumor-associated macrophages (TAMs), cancer-associated fibroblasts (CAFs), Tumor endothelial cells (TECs) and Tumor-associated adipocytes (TAAs). Created with BioRender.

Our group succeeded in designing a model of CSCs derived from induced pluripotent stem cells (iPSCs), which were reprogrammed from normal cells, in the conditioned medium from a variety of mouse and human cancer cell lines. These obtained CSCs exhibited a capacity of self-renewal, differentiation, and malignancy in vivo. Simultaneously, we demonstrated that CSCs are one of the key sources of CAFs, tumor endothelial cells (TECs), tumor-associated adipocytes (TAAs), and TAMs (Figure 1).

In this review, we summarize TME components discussing the origin of TME cells and the ability of CSCs to differentiate into tumor stromal cells, CAFs, TECs, TAAs, and TAMs providing the progenies, which establish a society in tumor tissue. We also discuss the potential contributions of CSCs in tumor progression, as well as an insight into cancer therapy.

## 2. The Tumor Microenvironment (TME)

**Figure 2:**
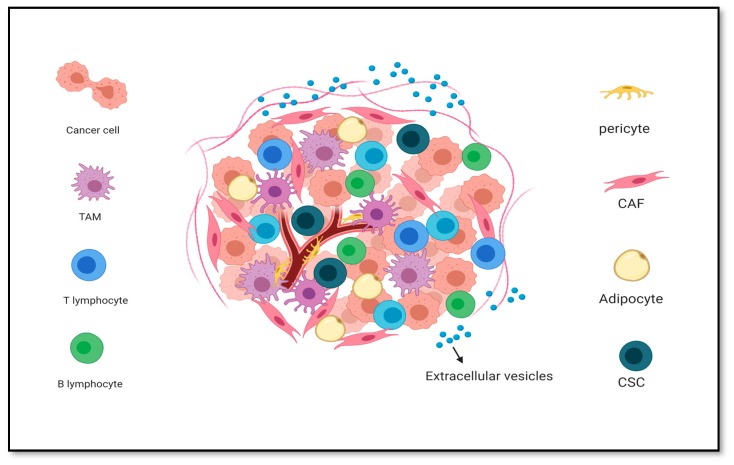
TME components, the different types of stromal cells, extracellular matrix (ECM), and extracellular vesicles. Created with BioRender.

The TME plays an important role in cancer development and progression. The TME is composed of two different cellular and acellular components [24]. The cellular component consists of tumor stromal cells, CAFs, TECs, pericytes, B lymphocytes, T lymphocytes, TAMs, TAAs and CSCs (Figure 2). The acellular component is composed of ECM, soluble factors and extracellular vesicles such as exosomes. Stromal cells, which form more than 80% of tumor bulk in pancreatic and breast cancers, are considered to play a vital role in the growth and progression of cancer [25]. Growth-promoting signals and intermediate metabolites secreted from the cellular component remodel the surrounding tissue structure and establish the TME [26]. The reciprocal communication between the cells in the microenvironment components eventually leads to enhanced proliferation and tumor metastatic capacity. Tumor cells need stromal cells to construct their microenvironment [23].

ECM provides a physical scaffold for all cells not only to reside in the TME but also to move in and out with a dynamic role causing the evolution and spread of cancers [27]. Cells are structurally and biochemically supported by an ECM, which is a scaffold of fibrillar proteins, accessory proteins, and molecules. The main component of the ECM is fibrillar collagen, the structure and mechanical properties of which have a strong influence on the cellular phenotype [28]. The ECM consists of a basement membrane and stroma, based on biochemical and structural characteristics. The basement membrane is largely made up of collagen IV in most tissues, along with laminin, fibronectin, and several proteoglycan forms. The basement membrane’s main role is to provide a physical barrier between the epithelial cells and the stroma of the organ, while still allowing the diffusion of gases and transport of signaling molecules. The interstitial ECM, mainly produced by mesenchymal cells, consists largely of collagens I and III, fibronectin, and proteoglycans. In cancer, the rupturing of the basement membrane enables epithelial cells to undergo an epithelial-to-mesenchymal transition (EMT) and migrate through the interstitial ECM into the surrounding stroma [29]. The ECM also contains key growth factors, like angiogenic factors and chemokines, which intermingle with cell surface receptors and provide that tissue with its tensile strength and elasticity, as well as its compressive strength. Tumors are harder than the surrounding normal tissues as a result of CAFs’ secretion of ECM components [30]. Large and rigid fibrils in the TME are caused by lysyl oxidase and transglutaminase enzymes that can cross-link collagen and elastin fibers [31]. On the other hand, malignant cells, TAMs and CAFs secret and stimulate matrix metalloproteases (MMPs), which degrade ECM proteins to promote TME remodeling [32].

Exosomes are cell-derived nanometer-size particles that have a key role in cell-to-cell communication. Most cells produce exosomes. Exosomes create the communication between the cells shuttling DNA, RNA, proteins and membrane-bound factors [33]. Tumor-derived exosomes (TEXs) affect the immediate TME. Furthermore, TEXs can affect distant tissues by the flow of blood and lymph to create a pre-metastatic niche that can lead to metastasis. Recent research demonstrated that TEXs were detected in the supernatant of cultured cells as well as body fluids. They can inhibit immune cell proliferation by delivering tolerogenic signals to immune cells. Intriguingly, TEXs exhibit certain ligands, like programmed death-ligand 1 (PD-L1), to generate an endocrine signal extending a distance away from the primary tumor [34]. Exosomes derived from CSCs induce tumor development and metastasis. CSC-derived exosomes significantly increase liver weight and serum levels of cancer markers, α-fetoprotein, and gamma-glutamyl transferase, as well as liver enzymes such as alanine aminotransferase, aspartate aminotransferase, and alkaline phosphatase. Severe immunostaining for glutathione S-transferase, an HCC marker, and a significant increase of number and area of tumor nodules were observed in rats received CSC-derived exosomes when compared to HCC [35]. CSC-derived exosomes also decreased apoptosis, increased angiogenesis, enhanced metastasis/invasion and induced EMT [36,37].

## 3. Differentiation of CSC Population in the TME

CSCs were first detected in acute myeloid lymphoma [38] and were then isolated in a variety of solid tumors including breast [39], colon [40], liver cancers [41], melanoma [42] and some other tumors. CSCs reveal several characteristics of embryonic stem cells and typically determine persistent activation of the Notch, Hedgehog, and Wnt pathways that are conserved in tissue development and homeostasis. CSCs maintain their self-renewal ability by activating several stem cell signaling pathways during cancer initiation and development while normal stem cells are involved in different developmental processes and tissue homeostasis. CSCs are considered to have decelerated growth rates and to be resistant to chemotherapy and radiotherapy [43].

In lung cancer, there is a rare population of undifferentiated tumorigenic cells expressing CD133, which is recognized as an antigen present in the cell membrane of normal and cancer-primitive cells of the hematopoietic, endothelial, neural and epithelial lineages [44]. Lung cancer CD133^+^ cells grew indefinitely as tumor-spheres in serum-free medium supplemented with epidermal growth factor and basic fibroblast growth factor (FGF-2). Once differentiated, these cells acquired the specific lineage markers while losing the tumorigenic potential as well as CD133 expression [45]. iPSCs have successfully been converted into CSCs with a conditioned medium of Lewis lung carcinoma cells [21], as well as those of other cancer cell lines [20,46]. Very recently, not only iPSCs but also embryonic stem cells have been converted to CSCs in the same manner [47]. The converted CSCs exhibited the potential of self-renewal, differentiation and malignant tumorigenicity with metastasis [48]. According to the CSC model, only certain subpopulations of cancer cells can drive the progression of cancer. They are more specific and aggressive subtypes of cells which could be responsible for tumor progression and recurrence [49].

The multidrug resistance (MDR) phenomenon means that resistance to therapy is not usually limited to one drug [50]. The MDR of CSCs is induced by the endogenous expression of detoxifying enzymes, increased DNA repair activity, increased pump levels of drug efflux, decreased drug response and activated survival pathways [51]. Various therapeutic strategies have been considered to target CSCs such as methods to target their cell-surface molecular markers, inhibit their self-renewal or therapeutic resistance-related differentiation pathways, modify their metabolism through glycolysis inhibition and mitochondrial regulation, as well as miRNA-based approaches to block CSCs [52]. New therapeutic methods have been developed using immunotherapy, anti-angiogenic compounds, and epigenetic trials to resolve CSC sensitivity to treatments [53].

CSCs can differentiate into various types of cells, including tumor cells and non-tumorigenic-differentiated cells in response to the specific stimulation of differentiation [54]. All-trans retinoic acid (ATRA), a carboxylic acid form of vitamin A, induced the differentiation of CSCs, increased sensitivity to therapies and reduced their motility and tumorigenicity by blocking angiogenesis in glioblastomas [55]. Treatment of breast CSCs with ATRA led to cell differentiation, diminished penetration and migration, and enhanced sensitivity to anticancer treatment [56]. Glioblastoma CSCs were capable of differentiation into mural-like endothelial cells [57]. As a result of CSCs’ differentiation, the cellular heterogeneity in tumors, as well as inherent drug resistance and invasive potential enhancement, plays a crucial role in tumor growth and metastatic progression [58]. Collectively, CSCs could be considered as a dynamic subpopulation of cancer cells with plasticity.

## 4. Cancer-Associated Fibroblasts (CAFs)

**Figure 3:**
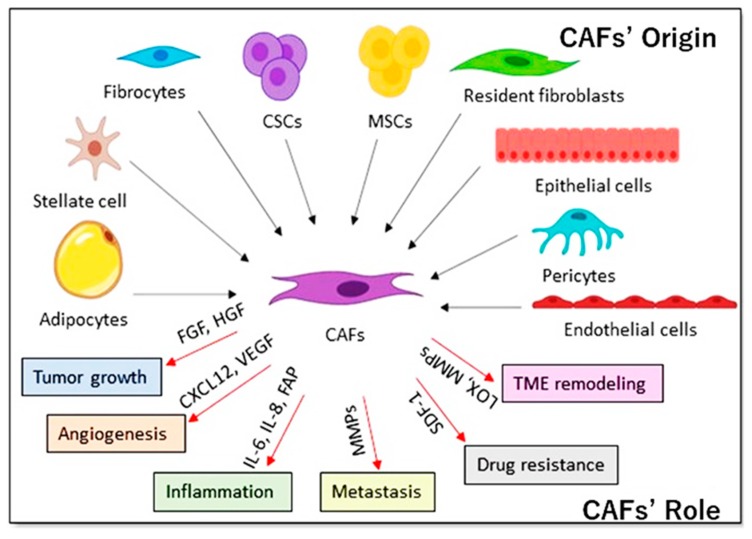
CAFs’ origin and role in the TME.

CAFs are considered to play a critical role in tumorigenesis by mediating tumor growth, inflammation, angiogenesis, stromal remodeling, metastasis and resistance to drug therapy (Figure 3). CAFs are the major TME component in many tumors [59], so they become the main target for suppressing tumor growth. The markers usually used to identify CAFs are α-smooth muscle actin (α-SMA) [60], platelet-derived growth factor receptor-β (PDGFR-β) [61], fibroblast-specific protein-1 (FSP-1) [62], and fibroblast activation protein-α (FAP-α) [63]. The phenotypic characteristics of CAFs are preserved even when cultured in the absence of interaction with cancer cells [64].

CAFs secrete growth factors, such as fibroblast growth factor (FGFs), insulin-like growth factor 1 (IGF1), hepatocyte growth factor (HGF) and members of the epidermal growth factor (EGF) family, which stimulate the growth of malignant cells [12,65] (Figure 3). CAFs also secrete transforming growth factor βs (TGFβs), which are considered to induce EMT in malignant cells and promote the immune-suppressive microenvironment [66]. Stromal cell-derived factor-1 (SDF-1)/CXC12 chemokine secreted from CAFs stimulates angiogenesis in breast cancer [12]. SDF-1 binds to CXCR4 facilitating the proliferation of lung cancer cell and drug resistance [67]. Simultaneously, CAFs secrete MMP-2 and membrane type 1-MMP (MT1-MMP), which are considered as the prerequisites for angiogenesis and metastasis in the carcinogenetic process [68].

Furthermore, CAFs secrete IL-6, IL-8, IL-4, and FAP that have vital roles in macrophage differentiation and polarization that lead to an immunosuppressive microenvironment. Tumor invasion and metastasis augmented by tumor necrosis factor (TNF) and IL-6 were identified as mast cell chemo-attractants [69]. IL-8 is associated with colorectal tumor size, infiltration, cancer stage, liver metastases, increased proliferation and migration of cancer cells [70,71]. TNF-α induces IL-8 expression in CAFs by the nuclear factor kappa B (NF-κB) activation [72].

A distinct ECM biomechanical architecture is required for tumor development and metastasis, ECM proteins are produced and secreted by CAFs that also actively participate in the ECM proteolysis, crosslinking and assembly processes [73]. One possible reason for tumor cells to escape from therapy and drug infiltration is a rigid and extremely crosslinked tumor stroma [74]. CAFs also mediate ECM remodeling by producing two main types of remodeling enzymes, the lysyl oxidase (LOX) family and MMPs. CAFs react to the ECM stiffness in a LOX/MMP-dependent way and fine-tune the CAF-ECM interactions as a highly adaptive and mechanically responsive stromal cell type [75,76]. Factors secreted from CAFs in the absence of serum/supplements strongly increased anchorage-independent growth, tumor-sphere formation, and expression of CSC-markers [77,78].

CAFs are morphologically like myofibroblasts and provide another TME pathway of ongoing support for the cancer niche. The stimulation of myofibroblasts can produce organ fibrosis which augments cancer growth [79]. Myofibroblasts are rich in many types of cancers and are as well-known as CAFs [64].

TEXs plays a crucial role in converting normal stromal cells to CAFs by TGF-β. CAFs are different from normal fibroblasts in many ways, such as their increased collagen and ECM protein construction and up-regulated secretion of pro-tumor factors [80]. The tumor stroma’s rigidity is affected by the interactions between CAFs and the ECM [81].

Many potential novel strategies for cancer therapy are considered to target tumor cells with genetic mutations rather than CAFs because of their genetic instability making them susceptible to therapeutic approaches [82]. On the other hand, retinoic acid receptor β expressed in CAFs makes strong chemoresistance, resulting in even complicated therapeutic responses of the cells [83]. CAFs also express cell-surface molecules CD10 and GPR77 that contribute to the chemoresistance supporting CSCs [84]. In addition, CAFs are activated by exposure to radiation, which induces the secretion of insulin-like growth factor-1 (IGF-1) from CAFs, resulting in making cancer cells resistant to such therapy. As a promising approach to restore the chemosensitivity in tumors, re-education of CAFs toward the normal fibroblasts by the epigenetic regulation of the dominant drivers responsible for CAF polarity is proposed with their inhibitors targeting the surface molecules on CAFs to suppress the secretomes [85,86].

CAFs may originate from populations other than resident fibroblasts depending on different mechanisms specific to tissues [87]. In breast, kidney, lung and liver carcinomas, a portion of CAFs has been displayed to potentially differentiate from epithelial cells via an EMT. The EMT was described to be engaged in the trans-differentiation of endothelial cells to a cell population with a phenotype like CAF’s [88]. The blood vessel-linked cells, termed pericytes, can trans-differentiate into CAFs in a platelet-derived growth factor (PDGF)-dependent manner [89].

Adipocytes were shown to differentiate into CAFs in breast cancer [90]. In liver and pancreatic tumors, stellate cells, while they are normally involved in organ regeneration, are a possible source of CAFs being involved in fibrosis preceding the occurrence of tumors [91,92]. Further than these local sources, distant cells can be implicated in the differentiation of CAFs in the TME. An important source of CAFs is mesenchymal stem cells that are normally found in the bone marrow but can be attracted to the TME [93]. Correspondingly, fibrocytes can differentiate into CAFs after their enrollment into the TME. They considered as a circulating inactive mesenchymal cell population arising from monocyte precursors which are recruited to the sites of chronic inflammation [94,95].

Furthermore, our group proved that CSCs converted from iPSCs could be the source of the CAFs which provide for tumor maintenance and persistence. We produced CSC-like cells by treating mouse iPSCs with conditioned medium from breast cancer cell lines [96]. CSC and pluripotency markers were expressed on the resulting cell population forming malignant tumors in vivo. The CSC-like cells isolated from the tumor have always developed heterogeneous population surrounded by cells like myofibroblasts. The cells displayed a CAF-like phenotype, suggesting that they had the potential to differentiate into another subpopulation of cells sustaining CSC self-renewal.

## 5. Tumor Endothelial Cells (TECs)

**Figure 4:**
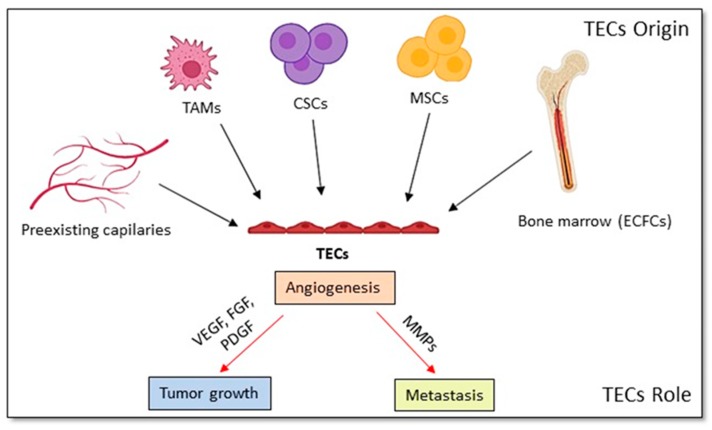
The origin of TECs and their roles in the TME.

New vessel development is a sign of tumor growth and progression (1–3). When a basic tumor mass (nearly 1–2 mm^3^) is formed, cancer cells require the promotion of angiogenesis with a tumor-associated neovasculature to efficiently supply nutrients and oxygen to themselves [97,98]. Tumor growth depends on angiogenesis. As all cells need to be close to the blood vessels that provide oxygen and nutrients, solid tumors cannot grow by more than a few millimeters in diameter without recruiting their own blood supply [99]. Tumor cells and various other cell types or the extracellular matrix in the tumor microenvironment release the endogenous molecules that affect the angiogenic balance. Angiogenesis stimulators include hypoxic conditions that activate the hypoxia-inducible factor alpha (HIF-1 alpha), which can upregulate angiogenic proteins, various growth factors such as vascular endothelial growth factor (VEGF), FGF and PDGF, as well as angiogenic oncogenes such as Ras. The effect of hypoxia on CSCs and their secretion of angiogenic factors resulting in tumor vascularization remains doubtful [100]. Tumor angiogenesis is considered to initiate from the growth of TECs stimulated by angiogenic factors, such as FGF, VEGF, and PDGF, including inflammatory cytokines secreted from tumor cells. As a result, MMPs and plasminogen activators are induced to cause the degradation of the vessel basement membrane allowing TECs to invade the surrounding tissues [101]. Then, the TECs deposit a new basement membrane and secrete growth factors, which will attract cells such as pericytes, ensuring the stable neovascular vessels to support the continued tumor growth. The TECs support the progression and metastasis of tumors [102,103] (Figure 4).

TECs, which have irregular shape and size, are completely different from normal endothelial cells (ECs). They have ruffled borders and long, fragile cytoplasmic processes extending outward and throughout the vessel lumen [104,105]. The tips of certain branched TECs could pierce the lumen, establishing openings or small intercellular gaps in the vessel wall. These openings permit extravagated erythrocytes to a pool of tumor blood vessels creating “blood lakes”. The appearance of tumor endothelium is defined as “mosaic” [106]. Only 0.1% to 3% of all ECs in normal tissues are estimated to turn over daily, and the percentage may decline with age. The rate of EC turnover in tumors is greatly accelerated 20–2000 times more than that in normal tissues [107,108].

Tumor angiogenesis has been considered to occur with the proliferating of endothelial cells in the blood vessels. Conversely, circulating endothelial progenitor cells CD34+/ VEGF receptor 2+ (VEGFR-2) were supposed to home into the areas of damaged tissue and encompass sites of active angiogenesis [109,110]. Other researchers identified similar cells, which were localized at angiogenesis sites within tumors circulating in blood [111]. Mesenchymal stem cells exhibiting endothelium formation in hemangioma are thought other sources for TECs [112]. Stem-like tumor cells could transdifferentiate to form endothelium as well [113]. Bone marrow MSCs may also be involved in tumor angiogenesis. MSCs can penetrate tumors and may augment cancer development [114] and under certain conditions, MSCs can differentiate into ECs [115,116]. ECs and MSCs appear able to transdifferentiate into each other being accelerated by the tumor microenvironment and contributing to tumor progression [117].

Several studies have shown that CSCs can support tumor angiogenesis and metastasis. CSCs may directly contribute to angiogenesis by differentiating into tumor vasculogenic stem/progenitor cells or creating a tumor microcirculation by developing vasculogenic mimicry devoid of an endothelial pattern [118,119]. CSCs were described to express angiogenic factors and alter elongated endothelial-like cells in vitro under hypoxia conditions [120]. CSCs were supposed to significantly transdifferentiate into ECs in tumor vasculogenesis. A population of tumor stem cells has been described to differentiate into TECs with the expression of the endothelial markers such as CD31, Factor VIII and, VEGFR2, acquiring the ability to form capillary-like structures after 6 h on Matrigel [121,122]. A model of CSCs induced from mouse iPSCs were demonstrated to differentiate into TECs [22]. The features of vasculature were evaluated in vivo showing neovascularization and vasculogenic mimicry formation [123]. CSCs’ subpopulation derived from mouse iPSCs was found to dominantly express angiogenic factors such as VEGF-A and FGF2. These results suggest that these CSCs have an important role in not only the enrollment of host endothelial vessels into tumor, but also in the development of endothelial linages with their progenies [124].

Pericytes are normally located on microvessel walls within a basement membrane opposed to the side of endothelium and typically recognized as extremely slender, elongated, and branched shape [125]. Pericytes were illustrated by the expression of α-SMA, platelet-derived growth factor receptor beta (PDGFRβ), desmin, CD146, and nerve/glial antigen-2 (NG2) proteoglycan [102]. The expression of α-SMA, CD146, PDGFRβ, and NG2 is not only limited to pericytes but varies depending on the type of tissue and the stage of maturation as well as the pathological conditions [126]. During the tumor progression, pericytes are considered to contribute to tumor angiogenesis, allowing endothelial cells to form vascular branches, surviving and circulating throughout the body [127]. However, little is known about the biology of these subpopulations of cells, nor about the exact origin of these cells or the development process. The basic functional roles of pericytes have long been recognized as maintenance and improvement of vasculature regulation of blood flow and vessel permeability [128]. Pericytes also essentially support ECs in a mechanical and physiological manner, which is critical for the vessel to remodel and mature [129]. Although, until now, the relationship between pericytes, stem cells, and CSCs has been obscure, pericytes are supposed to be comprised of stem cells of various cell types. Pericytes could be differentiated into chondrocytes and adipocytes [130]. Pericytes could also be a source of osteogenic cells [131,132].

## 6. Tumor-Associated Adipocytes (TAAs)

Adipocytes are the least studied stromal cells in all types of cancers, despite the fact that in some types, cancer cells are in direct connection with the adipocytes [133,134]. Adipose tissue is constituted of lipid-filled adipocytes and the stromal vascular fraction [135]. Monocytes/macrophages represent a large part of the stromal vascular fraction. Macrophages are remarkably plastic and can be assumed as multiple phenotypes [136]. Extra adiposity, namely obesity, is related to cancer risks, which is attributed to higher levels of pro-inflammatory factors secreted from the adipocytes chronically triggering wound healing. For instance, adipocyte-derived trophic factors (adipokines) could also promote the growth of tumors [137]. While adipose tissue-associated macrophages have been well described leaning against obese adipose depots [138], TAAs have not been studied in this regard. TAAs are supposed to serve as a depot of tumor-infiltrating activated macrophages, which might support tumor growth potentially activating neovascularization and exacerbating inflammation. Tumor cells modify adjacent TAAs by inducing inflammation, angiogenesis, and fibrosis [139,140]. TAAs in highly vascularized and angiogenic tumor tissues may support the expansion and progression of the tumors. These insights make tumors such as breast tumors, which are typically initiated by adipocytes within adipose tissues, feasible to grow directly or metastasize to lymph nodes [141,142].

In the case of caloric excess over a long time, adipocytes become hypertrophic and lose both metabolic activity and the control of pro-inflammatory cytokines, free fatty acids, and hormone liberation [143]. Dysfunctional adipose tissue is considered a hallmark for a chronic state of inflammation. The enhanced secretion of pro-inflammatory cytokines together with raised lipid metabolites promotes tumor progression [144]. Prominently, recent studies demonstrated that cancer cells and neighboring adipocytes in the tumoral stroma directly interact with each other [145]. This interaction will lead to activated adipocytes with tumor supportive phenotype, which is usually recognized by lipolysis, loss in adipocyte markers and overexpression of pro-inflammatory cytokines [146]. Adipocytes in breast TME also enhance tumor cell survival, increasing resistance to chemotherapy. The proliferation and invasion of tumor cells into adjacent tissues could be attributed to the roles of TAAs within the tumor tissue, while the origin of TAAs remains unclear [147].

Our group previously reported that CSCs converted from iPSCs could arise from normal stem cells when treated in the cancer microenvironment [21], including tumor-derived extracellular vesicles, which were secreted from lung cancer-derived cells. Interestingly, the CSCs generated by the treatment with the vesicles from mouse iPSCs exhibited malignant liposarcomas with aggressive dissemination into the abdominal cavity in vivo. The CSCs established from primary tumors showed the differentiation potential to adipocytes as well as the capacity of self-renewal. These results suggest that CSCs could be the origin of TAAs in some cases [148,149].

## 7. Tumor-Associated Macrophages (TAMs)

Macrophages are the most abundant immune cells in the TME. Macrophages are considered to have immunologic tumoricidal activity [150] while they adopt a pro-tumoral phenotype both in primary and metastatic sites [151].

As one of the types of immune cells in the TME, macrophages have been considered to play an important role in tumor progression depending on the stage of tumor growth and the type of tumor [152]. TAMs are tolerated through various mechanisms to enhance tumor progression. Conventionally, macrophages are classified into two subgroups of M1 and M2. M1-type macrophages are classically defined as ones activated by pathogens and innately fighting against invading pathogens [153]. Alternatively, M2-type macrophages are active macrophages that play important roles in tissue repair or tumor progression. Many studies have shown that Notch signaling plays a crucial role in the polarization of M2 macrophages [154]. A previous study showed that the transcription factor Gata-6, which plays a key role in fixing the phenotype of macrophages by altering their transcriptome, can be expressed specifically by mouse peritoneal macrophages and renewing macrophages in the inflammatory response [155]. Several cytokines, such as IL-6 have found to be involved in the development and preservation of the macrophage subsets and their functions in inflammation and tissue homeostasis [156]. Many soluble factors responsible for the polarization of macrophage-supporting tumor progression have been identified. Monocytes enroll in the tumor tissue as a result of chemokine CCL2 and macrophage colony-stimulating factor [157]. Interleukins such as IL-4, -10 and -13, as well as other cytokines in the TME, stimulate the differentiation of monocytes into TAMs [158]. Although the ability to present antigens is very weak, TAM is considered to have the potential to promote tumor progression through a variety of mechanisms, which are not known in detail. In lung cancer, macrophages are found polarized to a pro-tumoral phenotype at the time of tumor initiation [159]. These activities include the suppression of T cell responses [160]. Also, macrophages facilitate many essential tumor progression features including angiogenesis, invasion of tumor cells, motility, tumor cell extravasation enhancement and persistent growth [161]. Each of these behaviors is provided by an identifiable subpopulation of macrophages. Immune cell commitment by tumors is necessary for their acquisition of a malignant phenotype. This argument came from data mentioned above together with previous studies showing that the ablation of macrophages inhibits tumor progression and metastasis. TAMs, therefore, could be an important therapeutic target for cancer treatment [162,163].

**Figure 5:**
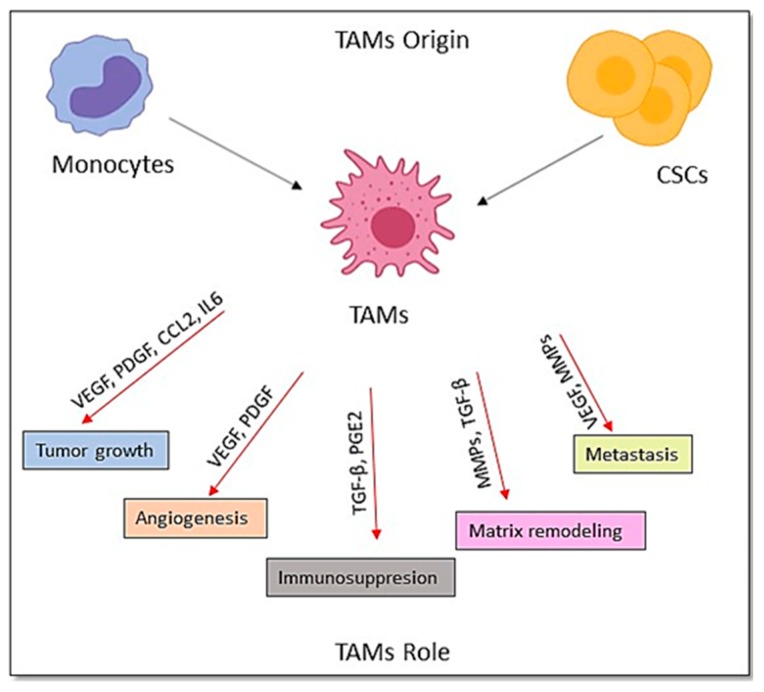
The origin and the roles of TAMs in the TME.

TAMs secrete a huge number of angiogenic factors, such as VEGF and PDGF (Figure 5), which can stimulate tumor angiogenesis [164]. TAMs also secrete various growth factors, MMPs, which promote tumor cell proliferation, invasion and metastasis. Furthermore, TAM has recently been found to express PD-L1 directly inducing T cell apoptosis [165]. TAMs are also considered to inhibit T cell growth by diminishing the local concentration of arginine, which is an essential amino acid for T cell metabolism to survive [166]. Being adopted by tumors, macrophages with an M2-like phenotype decrease their MHC class II molecules and become ineffective antigen-presenting cells [167].

The high density of TAM correlates with therapeutic resistance and poor prognosis of cancer patients. Hence, limiting tumor growth and metastasis as well as restoring chemotherapeutic responsiveness have been successful after macrophage depletion [168]. For example, Trabectedin, which is a DNA-binding agent, applies selective cytotoxicity to circulating monocytes and TAM populations by stimulating the extrinsic apoptotic pathway depending on the tumor necrosis factor [TNF]-related apoptosis-inducing ligand (TRAIL). In particular, monocytes are sensitive to TRAIL because they express very minimal levels of TRAIL decoy receptors [169]. The production of cytokines, including CCL2 and IL-6, which are important in promoting tumor growth, is significantly inhibited by trabectedin in four different mouse tumor models. TAMs produce CCL18 and stimulate the invasiveness of breast cancer cells via phosphatidylinositol transfer protein membrane-associated 3 [170,171].

The origins of macrophages are still elusive in many cancers, especially in the early stages [172]. The recruitment and differentiation are likely to be different from those when exposed to microbial products and more complex in cancers, especially in colon cancer, than those in acute inflammation. Nevertheless, the possibility of therapies targeting the pro-tumoral macrophages that spare the resident macrophages associated with homeostasis from anti-tumoral activities is tantalizing, because the investigation of the origins of TAMs and their measures of recruitment, maintenance, and differentiation make up the primary stages of them [173,174].

The historic concept of adult resident tissue macrophages as exclusively derived from bone marrow (BM) has recently been proved incorrect. Most tissue macrophages arise from yolk sac progenitors, with some exceptions such as those from intestines. In contrast, macrophages responding to pathogens appear to come from circulating BM-derived monocytes [175,176]. Several embryonic origins contradict the belief that TAMs derive from the BM in the primary tumor. Recently, evidence of different origins and responses with the presence of TAMs as resident yolk sac-derived microglia and recruited BM-derived monocytes has been shown in a mouse model of glioma. TAMs in the TME have different behavior to anti-macrophage treatments based on the inhibition of colony-stimulating factor-1 signaling, which regulates the lineage [151,177].

CSCs could be the source of TAMs in the TME. Our group has recently analyzed a malignant tumor developed from CSCs converted from human iPSCs. High immunoreactivity to both anti-human and anti-mouse CD68 antibodies was detected, suggesting that the tumor tissue was enriched by TAMs originated from both human and mouse cells.

In mouse models, pharmacological macrophage inhibition has shown great promise and several agents are currently under clinical investigation. Macrophage depletion and recruitment should be included in several strategies targeting macrophages within the TME [157,178].

## 8. CSCs: Challenges and Limitations

There is still no precise information on the differentiation abilities of CSCs even though they have shown pivotal characters as a subpopulation of cancer cells, such as chemoresistance and metastatic ability. The definition of CSCs suggests that these cells have also differentiation ability as well as normal stem cells. This raises a question— “What are the boundaries of this ability?”.

Major obstacles must be overcome to comprehend resultant cell phenotypes and mechanisms of differentiation. Identification and isolation of CSCs are two of these obstacles. To isolate CSCs, antibodies against specific surface markers or specific culture conditions should be used that why until now obtaining CSCs is still considered a challenging and demanding procedure. At the same time, genetic manipulation or reprogramming cells into CSCs is ignoring the epigenetic effects on inducing CSCs. To get through this issue, our lab developed a novel method for converting iPSCs into CSCs. The conversion was accompanied by tumorigenicity acquiring, elevating CSC markers and maintaining stemness markers. Interestingly, the number of hypomethylation of CpG islands in the converted CSCs was found more than that in iPSCs [179]. This observation suggested CSCs were highly plastic having potentially active genes more than iPSCs did. As mentioned above, using these models, we have proved that multiple phenotypes could be differentiated from CSCs converting from iPSCs. These differentiated cells have also shown supportive roles in maintaining CSCs.

Studying CSCs converted from iPSCs to identify different subtypes of CSCs and their heterogeneous plasticity could be helpful to establish new methods for isolation and expansion of patient-derived CSCs. Still needed to explore is their relevance to patient-derived CSCs although the importance of CSCs converted from iPSCs in the cancer research field has been conveyed.

In the meantime, the fundamental question remains if patient-derived CSCs or cancer cells are exhibiting the same plasticity as CSCs converted from iPSCs and what is the difference between their plasticity. A part of this question has come to light since several studies from different groups have proved the ability of bulk cancer cells or cancer cells with stemness characteristics for differentiation into other cell phenotypes. Xenografts of glioma stem cells have shown to contain vessels of human origin. Glioma and colorectal cancer stem cells had displayed the ability to differentiate into endothelial and smooth muscle-like cells [122,180,181,182,183,184]. These results coincide with our results which showed the ability of CSCs converted from iPSCs to differentiate into endothelial cells with the ability of tube formation [123]. Regarding blood and immune cells, many studies have shown that polyploidy giant cancer cells with the expression of stemness markers can differentiate into myoepithelial, endothelial and erythroid cells with a marker of hemoglobin [185,186,187,188,189]. This is in particular interesting because the well-believed concept of the bone marrow origin of TAMs have considerably changed recently. TAMs are now considered to have embryonic origin rather than bone marrow origin [190,191]. This fact is raising a question on the possibility of TAMs having CSC origin. Finally, breast cancer cells have differentiated into functional adipocytes which also prove the transdifferentiation ability of cancer cells [192,193,194].

In the summary, the CSCs converting from iPSCs have proposed some differentiation patterns of CSCs, which were further confirmed using other types of cancer cells and CSCs. Thus, exploring the roles of different cell phenotypes arising from CSCs in the TME could provide more efficient treatment strategies and drug combinations for cancer in the future.

Although there are increasing studies that examine the plasticity of CSCs, there is still a lot of information missing regarding mechanisms, factors, and conditions that drive CSCs to give other types of cells in the TME. Thus, further studies are needed to evaluate the plasticity of CSCs converted from iPSCs, CSCs derived from cell lines and patient-derived CSCs. Correspondingly, it appears very important to identify different stages of plasticity in CSCs and ones to be targeted so that the differentiation cascade could be stopped to provide progenies.

## 9. Conclusions

Here, we described our hypothetical view of the differentiation ability of CSCs into some types of cells in the TME which may support CSCs and tumor growth based on our results obtained by the CSCs converted from iPSCs. At the same time, we discussed some recent studies that proved the same idea using CSCs from cancer cell lines. While many cell phenotypes are still not explored yet, we believe that this review could prompt more discussion in the community of cancer science about the potential of CSCs to construct their own niche by differentiating into other cell phenotypes.

## Abbreviations

**Abbreviations**	**Full Names**
ATRA	all-trans retinoic acid
BM	bone marrow
CAFs	cancer-associated fibroblasts
CSCs	cancer stem cells
ECM	extracellular matrix; ECs: endothelial cells
EMT	epithelial mesenchymal transition
FAP-α	fibroblast activation protein-α
FGF-2	fibroblast growth factor
FGFs	fibroblast growth factor
FSP-1	fibroblast-specific protein-1
HGF	hepatocyte growth factor
HIF	hypoxia-inducible factor
IGF1	insulin-like growth factor 1
LOX	lysyl oxidase
MDR	multidrug resistance
MMPs	matrix metalloproteases
MSCs	mesenchymal stem cells
MT1-MMP	membrane type 1-MMP
NF-κB	nuclear factor kappa B
NG2	nerve/glial antigen-2
PD-L1	programmed death-ligand 1
PDGF	platelet-derived growth factor
PDGFR-β	platelet-derived growth factor receptor-β
SDF-1	Stromal cell-derived factor-1
TAAs	tumor-associated adipocytes
TAMs	tumor-associated macrophages
TECs	tumor endothelial cells
TEX	tumor-derived exosomes
TGFβs	transforming growth factor βs
TME	tumor microenvironment
TNF	tumor necrosis factor
TRAIL	TNF-related apoptosis-inducing ligand
VEGF	vascular endothelial growth factor
VEGFR-2	VEGF receptor 2
iPSCs	induced pluripotent stem cells
α-SMA	alpha-smooth muscle actin.
